# Functions of miRNAs during Mammalian Heart Development

**DOI:** 10.3390/ijms17050789

**Published:** 2016-05-21

**Authors:** Shun Yan, Kai Jiao

**Affiliations:** Department of Genetics, the University of Alabama at Birmingham, Birmingham, AL 35294, USA; syan@uab.edu

**Keywords:** miRNA, *Dicer1*, cardiogenesis

## Abstract

MicroRNAs (miRNAs) play essential roles during mammalian heart development and have emerged as attractive therapeutic targets for cardiovascular diseases. The mammalian embryonic heart is mainly derived from four major cell types during development. These include cardiomyocytes, endocardial cells, epicardial cells, and neural crest cells. Recent data have identified various miRNAs as critical regulators of the proper differentiation, proliferation, and survival of these cell types. In this review, we briefly introduce the contemporary understanding of mammalian cardiac development. We also focus on recent developments in the field of cardiac miRNAs and their functions during the development of different cell types.

## 1. Contemporary Understanding of Heart Development

The heart is the first organ to develop and function in mammals, and its normal formation is essential for fetal life. During early gastrulation, cardiac progenitor cells within the anterior lateral plate mesoderm migrate to the cranial and cranio-lateral regions of the embryo. There are two distinct populations (or cardiac fields) of progenitor mesodermal cells that contribute to the developing heart: the first heart field (FHF) and the second heart field (SHF) [1,2,3]. The FHF originates from cells in the anterior splanchnic mesoderm and forms the cardiac crescent at approximately embryonic day 7.5 (E7.5) in mice [1,4]. These cells move toward the ventral midline of the embryo; by E8.0, they coalesce to form an early heart tube. The early heart tube consists of an exterior myocardial layer and an interior endocardial layer, which are separated by the extracellular matrix (ECM) [5]. At about the same stage, cardiac precursor cells within the SFH start to join the heart tube to its arterial and venous poles from the pharyngeal mesoderm and the dorsal mesocardium. Starting at approximately E8.5 in mice, the heart tube loops to the right to position the atria above the ventricles. Regional proliferation along the myocardium of the outer curvature of the looping heart contributes to the formation of the future atrial and ventricular chambers. The cardiac precursor cells from the SHF continue to join the heart until approximately E10.5-11.5. In a four-chamber heart, FHF-derived cells give rise to the left ventricle as well as parts of the atrial chambers. SHF-derived cells contribute to the main parts of the atrial tissue, the right ventricle, and the outflow tract (OFT) [1,2,3,5,6]. Embryonic cardiomyocytes continuously undergo hyperplastic growth and maturation. Shortly after birth, their proliferation capacity is mostly lost and cardiac growth depends almost exclusively on hypertrophic growth.

Between E9.5 and E11.5 in mouse embryos, a subset of endocardial cells in the atrioventricular canal and OFT regions respond to signals from the overlying myocardium and undergo epithelial-to-mesenchymal transition (EMT) to form mesenchymal cells in endocardial cushions [7,8,9,10,11]. Cellularized cushions act as the primordia of valves and septa to facilitate unidirectional blood flow in embryonic hearts. During the later developmental stages that start at approximately E11.0 in mice, these cushions undergo complicated remodeling processes to become the mature valvar and septum structures between the atria and ventricles and between the OFT arteries and ventricles. Endocardial cells and their derivatives are the major source of valvar cells.

Two additional cell populations—neural crest cells (NCCs) and epicardial cells—are also critical for normal heart development. Cardiac NCCs are derived from the neural folds between the mid-otic placodes and the caudal limit of somite 3. They migrate through pharyngeal arches and join the distal region of the OFT cushions to facilitate the separation of the aorta root and the pulmonary trunk [12,13]. NCCs give rise to the smooth muscle cells of the pharyngeal arteries and to a sub-group of the interstitial cells in the semi-lunar valves. The epicardium is derived from a transient structure called the proepicardium organ (PEO) that forms near the sinus venosus at about E9.5 in mice. During heart looping, PEO cells adhere to and spread over the myocardium to form the primitive epicardium. By E11.0, a sub-population of epicardial cells undergo EMT to invade the myocardium and differentiate into cardiac fibroblasts and coronary smooth muscle cells [14,15].

Errors in cardiac development lead to congenital heart diseases (CHDs). CHDs are the most prevalent human birth defect, affecting approximately 1%–5% of newborns [16,17,18,19]. Accumulating evidence supports the idea that microRNAs (miRNAs) are essential for the proper regulation of gene expression during mammalian heart development [20,21,22,23,24,25]. In the following section, we will review the current knowledge regarding the functions of miRNAs in the developing heart.

## 2. Functions of miRNAs during Cardiac Development

### 2.1. Regulation of Cardiomyocyte Development by miRNAs

Among the four major cell types, the functions of miRNAs in developing cardiomyocytes have been the most studied. The specific deletion of *Dicer1* in embryonic cardiomyocytes using different Cre drivers has been applied to examine the role of global miRNA biosynthesis in these cells. The deletion of *Dicer1* in cardiac progenitor cells using an *Nkx2.5-Cre* driver leads to delayed ventricular development, pericardial edema, and embryonic death at E12.5 [26]. The deletion of *Dicer1* using another *Nkx2.5-Cre* driver that inactivates targets with subtly different spatiotemporal kinetics leads to the double-outlet-right-ventricle defect, suggesting that miRNAs play a role in regulating the alignment of the major arteries and ventricles [27]. The myocardial deletion of *Dicer1* at the mid-gestation stage using the *cTnt-Cre* line leads to embryonic lethality at E15.5 [28]. Mutant hearts display severe myocardial wall defects including reduced cell proliferation, increased cell death, and non-compaction. The myocardial inactivation of *Dicer1* during late gestation using the *MHC-Cre* driver results in dilated cardiomyopathy, heart failure, and lethality within four days of birth [29]. These data collectively indicate that miRNAs have complex roles at different cardiogenic stages.

In addition to the above research regarding global miRNA biosynthesis, many miRNAs have been shown to be critical for cardiomyocyte development. miR-1 and miR-133 are two highly conserved miRNAs that exhibit cardiac- and skeletal muscle-specific expression during development and in adults [30,31]. miR-1 negatively regulates cardiac growth and differentiation by inhibiting the translation of the transcription factor HAND2 [30]. The level of HAND2 is critical for ventricular cardiomyocyte expansion [30,32,33,34]. Mice lacking miR-1-2 show defects in cardiac morphogenesis, cardiac conduction, and ventricular hypoplasia [26]. In mice overexpressing miR-1, the number of proliferating cardiomyocytes is decreased [35]. Similarly, miR-133 negatively regulates cardiomyocyte proliferation by inhibiting its targets Cyclin D2 and Serum Response Factor (SRF) [36]. Mice with a deletion of either miR-133a-1 or miR-133a-2 are phenotypically normal, suggesting that miR-133a-1 and miR-133a-2 perform redundant roles during heart development. However, miR-133a-1/miR-133a2 double mutant mice exhibit ventricular-septal defects and embryonic lethality, while the surviving null mutant mice display dilated cardiomyopathy and heart failure [36]. In contrast, the overexpression of miR-133 in embryonic cardiomyocytes causes embryonic lethality due to reduced cardiomyocyte proliferation [36].

In addition to the two most abundantly expressed miRNAs in the heart, multiple other miRNAs have been reported to regulate cardiomyocyte proliferation and apoptosis. These include the miR-17-92 cluster, the miR-15 family, and miR-590, miR-199a, miR-320, and miR-98/-128/-142 [22,23,28]. miR-17-92 promotes cardiomyocyte proliferation in embryonic, postnatal, and adult hearts by negatively regulating the tumor suppressor PTEN [37]. Mice that specifically overexpress miR-17-92 in cardiac cells exhibit an increased number of cardiomyocytes and more strongly protect the heart from myocardial infarction-induced injury [37]. Members of the miR-15 family (miR-195, miR-15a, miR-15b, miR-16, and miR-497) have been shown to inhibit cardiomyocyte proliferation by repressing multiple cell cycle regulators [38] and to induce apoptosis by targeting the anti-apoptotic factor *Bcl2* [39]. In particular, the overexpression of miR-195 in embryonic or postnatal mouse hearts leads to cardiac hypertrophy and heart failure [38,40]. miR-590 and miR-199a can promote cardiomyocyte proliferation in neonatal and adult rodent hearts [41]. miR-320 has been demonstrated to induce cardiomyocyte apoptosis by decreasing expression of heat-shock protein 20, a cardio-protective protein, and thus is involved in the regulation of ischemia/reperfusion (I/R)-induced cardiac injury [42]. miR-98, miR-128, and miR-142 directly target *Tgfbr1* mRNA to repress TGFβ activity in the developing myocardium [28]. Aberrant elevations in TGFβ activity impair cardiomyocyte proliferation and survival in mouse embryos [28,43].

Several miRNAs regulate the expression of cardiac myosin genes. In rodents, there are two myosin isoforms that are expressed in the heart—αMHC (MYH6) and βMHC (MYH7). βMHC, the slow ATPase, is predominantly expressed in cardiomyocytes prior to birth, while αMHC, the fast ATPase, is highly expressed in the adult heart [44]. The myomiR family (miR-208a, miR-208b, and miR-499) is involved in reactivating the cardiac fetal gene program when the heart is exposed to cardiac stress conditions or in response to hypothyroidism [45,46,47]. miR-208a, miR-208b, and miR-499 are encoded within the introns of the *Myh6*, *Myh7*, and *Myh7b* genes, respectively [45]. The expression of miR-208a and miR-208b parallels the expression of their respective host genes during normal heart development and in diseased hearts [46]. miR-208 and miR-499 function in the regulation of myosin expression by controlling the activity of their downstream targets, which include *Thrap1* and several transcriptional repressors of *Myh7* [46,47]. miR-208a-null mice show no obvious phenotype, suggesting that miR-208a is not essential for cardiac development [46,47]. However, mice lacking miR-208a are resistant to cardiac hypertrophy and fibrosis induced by the transgenic expression of calcineurin, indicating that miR-208a is indispensable for the cardiac stress response [47]. Consistently, the overexpression of miR-208a in mice is sufficient to induce cardiac hypertrophy [46].

The miRNA-17-92 cluster promotes SHF myocardial differentiation. miR-17-92 mutant mice die shortly after birth due to a combination of double-outlet-right-ventricle and ventricular-septal defects [48,49]. Bone Morphogenetic Protein (BMP) signaling promotes the transcription of the miR-17-92 complex during myocardial differentiation in the SHF via SMADs. The simultaneous inactivation of *Bmp2* and *Bmp4* in the SHF upon using the *Mef2c-Ahf-Cre* driver significantly reduces the expression of multiple miR-17-92 miRNAs and impairs myocardial differentiation [49]. The miRNA-17-92 complex promotes SHF myocardial differentiation by directly repressing the cardiac progenitor genes *Isl1* and *Tbx1* [49]. miR-17-92 functional seed target sequences are present within the 3′-untranslated regions of *Isl1* and *Tbx1.* In *Bmp2/4*-mutant embryos or miRNA-17-92-null embryos, the expression of *Isl1* and *Tbx1* is upregulated, leading to the inhibition of cardiomyocyte differentiation. In contrast, the transgenic overexpression of miR-17-92 in mouse embryos downregulates the expression of *Isl1* and *Tbx1* [49].

### 2.2. Functions of miRNAs in Cardiac Neural Crest Cells (NCCs)

miRNA biosynthesis is essential for cardiac NCC development. The NCC-specific deletion of *Dicer1* in mice leads to multiple cardiovascular defects including an interrupted aortic arch and double-outlet-right-ventricle and ventricular-septal defects, which phenocopy certain forms of human congenital cardiac defects [50,51,52,53]. Similar defects are observed in mice with a NCC-specific inactivation of *Dgcr8*, which encodes an essential cofactor for Drosha [54]. The inhibition of miRNA biosynthesis does not appear to inhibit the migration of cardiac NCCs to the OFT. Rather, it impairs the normal survival and differentiation of NCC derivatives after NCCs migrate to their target sites. Interestingly, the increased cell apoptosis in the pharyngeal arch of mutant embryos is not limited to NCCs, but is also found in the surrounding mesoderm-derived mesenchymal cells. This supports a potential role for NCC-expressed miRNAs in mediating the interaction between NCCs and their neighboring cells [53].

Multiple miRNAs can regulate the differentiation of NCCs into smooth muscle cells. The ectopic expression of miR-145 is sufficient to induce the differentiation of multipotent neural crest stem cells into vascular smooth muscle cells as demonstrated by the expression of vascular smooth muscle cell (VSMC) differentiation genes [55]. Furthermore, miR-145 can potentiate the Myocardin-dependent reprogramming of adult fibroblasts into VSMCs [55]. miR-143 and miR-145 work together to regulate the differentiation and proliferation of VSMCs by targeting a network of transcription factors that includes *Klf4*, Myocardin, and *Elk1*. In cultured VSMCs, the expression of miR-143/145 is downregulated during cell dedifferentiation. Conversely, the induction of miR-143/145 enhances the differentiation of VSMCs and represses their proliferation [55,56]. These results indicate that miR-143/145 may play essential roles in controlling the phenotypic switching of SMCs. Mechanistically, miR-143 targets *Elk1* (encoding an activator of VSMC proliferation), and miR-145 targets Myocardin (encoding an activator of VSMC differentiation) as well as *Klf4* and calmodulin kinase II-Δ (positive regulators of cell proliferation). miR-143/145 double-knockout mice are viable and do not exhibit any obvious abnormalities. However, the postnatal mice are hypotensive due to the reduced tunica media of the aorta and femoral artery [55,57,58].

### 2.3. Functions of miRNAs in Epicardial and Endocardial Cells

The roles of miRNAs during epicardial and endocardial development have not been thoroughly studied. In the epicardium, *Dicer1* is required for the development of the coronary vasculature [59]. Mice with an epicardial-specific deletion of *Dicer1* die immediately after birth, exhibit coronary vasculature defects due to an impaired EMT, and display reduced epicardial cell proliferation and differentiation [59]. In these mutant hearts, the EMT of epicardial cell is severely affected, and the expression of EMT regulators is altered (the expression of *Wt1*, *Snail1*, *Snail2*, *and* *Twist1* is reduced, and the expression of E-cadherin is increased). *Dicer1* mutants also show a significant downregulation of epicardial cell proliferation and failed smooth muscle differentiation in the developing coronary vasculature [59]. miRNAs that regulate the EMT, including miR-21, miR-31, miR-103/107, miR-155, and the miR-200 family, have been implicated in playing important roles during epicardial development [59,60,61]. Specifically, miR-21 positively modulates fibrogenic EMT in epicardial mesothelial cells (EMCs) by targeting Programmed Cell Death 4 (PDCD4) and Sprouty Homolog 1 (SPRY1) [60]. The overexpression of miR-21 in non-stimulated EMCs leads to a reduction in the expression of the mesenchymal biomarker E-cadherin, while the knockdown of endogenous miR-21 during TGFβ-induced EMT promotes the expression of E-cadherin [60]. In contrast to miR-21, miR-31 negatively regulates fibrogenic EMT in EMCs by directly repressing the cardiac progenitor gene *Isl1*. In non-stimulated EMCs, ISL1 promotes mesenchymal features. In TGFβ-induced EMCs, miR-31 reduces ISL1 and thereby inhibits the EMT [61].

The functions of miRNAs in endocardial cells have mainly been studied in zebrafish. miR-23 and miR-218 are involved in endocardial cell differentiation and migration in zebrafish [62,63]. miR-23 restricts endocardial cushion formation by inhibiting its direct target *Has2* and the extracellular production of hyaluronic acid. miR-23 loss-of-function leads to excessive endocardial cushion cell differentiation in zebrafish embryonic hearts [62]. Consistently, in mouse endothelial cells, miR-23 inhibits TGFβ-induced EMT [62]. miR-218 regulates the formation of the linear heart tube in zebrafish by negatively modulating Slit/Robo signaling during heart field migration. The knockdown of miR-218 delays heart field fusion and reduces the endocardial migration rate [63].

## 3. Summary

In summary, the regulation of cardiogenic gene expression by miRNAs plays indispensable roles in mammalian cardiogenesis. A better understanding of how miRNAs act during heart development in different cell types will greatly advance our fundamental knowledge regarding the molecular mechanism underlying cardiogenesis and is essential for the development of novel diagnostic and therapeutic strategies for congenital and acquired heart diseases in human patients. Based on the functions of endogenous miRNAs in different cell types, we can design miRNA mimics and/or antagomiRNAs to modulate the activity of specific miRNAs to treat various cardiovascular diseases. The effectiveness of this approach has been demonstrated in animal models with myocardial hypertrophy, heart failure, and cardiac fibrosis [64,65].
